# Dr. M. O. Heydock

**Published:** 1881-05

**Authors:** 


### Obituary.

---

IN MEMORY OF DR. M. O. HEYDOCK.—Resolutions offered at the meeting of the Chicago Medical Society, April 18, 1881, by the committee appointed at that meeting, relative to the death of Dr. M. O. Heydock:

*Resolved*, That in the death of Mills Olcott Heydock, M.D., on the morning of the 17th inst., after a brief and severe illness, this Society has lost one of its most esteemed members, the profession one of its most enlightened and useful practitioners, and society a most valuable citizen.

*Resolved*, That we deeply sympathize with the widow and relatives in their severe affliction, and commend them for consolation to the great Giver of all good, who alone can heal the wounded spirit.

N. S. DAVIS,
WM. E. CLARKE,
R. G. BOGUE.

Per L. H. MONTGOMERY, *Secretary*.

---

EULOGIUM BY DR. JOHN BARTLETT.—*Mr. President:* It is a painful occasion to us all to-night, when are to be uttered the last kindly words in remembrance of our late fellow, Dr. Heydock. It is an occasion especially sad to those of us, who by concurrence of age, and period of residence here, have the more intimately associated with him who has so suddenly been called hence.

Mr. President, while words of sorrow at the departure of a loved friend are so faintly expressive of one's feeling as almost to prepare the silence which grief invites, yet as the stricken heart of man can but seek relief in utterance, something needs be said ere the name of M. O. Heydock fades from our lists. To a natural adaptation for his profession, Dr. Heydock brought the advantage of a superior intellect, well trained by the mental athletics incident to the acquirement of a collegiate education. To these qualities were superadded in Dr. Heydock a genial disposition, coupled with an urbanity and pleasing dignity of manner, which ever made his presence welcome. Mr. President, I have known our departed friend for twenty years, and I have ever known him as an earnest, methodical student, a painstaking, conscientious practitioner, and an honorable colleague. He was all that is implied in these words, "A scientific physician and a Christian gentlemen."

Bereft of the presence of our associate, we have yet remaining the pleasant assurance that so long as memory endures, we can never forget the example he has set for us, of faithful work and honorable conduct.

